# Tackling local ecological homogeneity: Finding intraspecific trait variability in local populations of Mediterranean plants

**DOI:** 10.1002/ece3.10550

**Published:** 2023-09-19

**Authors:** Lorenzo Maria Iozia, Laura Varone

**Affiliations:** ^1^ Department of Environmental Biology Sapienza University of Rome Rome Italy

**Keywords:** aridity, drought, environmental variability, intraspecific variability, local homogeneity, plant functional traits

## Abstract

Local homogeneity, in ecology, is the often undisclosed assumption that variability within populations is negligible or mostly distributed evenly. In large areas, this can lead to the aggregation of different populations without regard for their unique needs and characteristics, such as drought sensitivity and functional trait distributions. Here, we discuss whether this assumption can be justified, and we hypothesize that discerning the source of variation between plasticity and adaptation could be a feasible approach to formulate an informed decision. We test this hypothesis on plants, resorting to a common garden experiment to determine the source of variation of several plant functional traits at a local scale (~60 km) of three wild species: *Quercus ilex*, *Pistacia lentiscus*, and *Cistus salviifolius*. Individuals of each species were sourced from three key sites chosen along a local aridity gradient. Our approach led to the rejection of the local homogeneity assumption for *Q. ilex* and *C. salviifolius* at this scale due to the adaptive divergence observed among neighboring populations. This case study provides evidence that addressing local homogeneity can highlight diverging populations in a relatively simple way. We conclude that gathering empirical evidence on intraspecific variability is a feasible approach that can provide researchers with solid bases to decide whether to adopt the local homogeneity assumption or not.

## Graphical abstract

Although the decision of grouping together individuals to define a population is usually arbitrary, we believe this choice to be based on the assumption that these individuals will share common features (local homogeneity). With this paper, we provide our insights regarding this assumption, and offer a more solid approach in defense of these choices, by discerning the source of variation within the considered group between phenotypic plasticity and adaptation. We accompany this idea with a case study, providing an example of this framework being used.

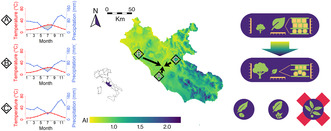

## 1 INTRODUCTION

In the ecological context, local homogeneity is the idea that organisms from close places must share common features. Such features could include functional traits (Diaz et al., 1998), ecological niches (Wagner & Fortin, 2005), and functional responses to environmental stress (Moran et al., 2016). This is a common assumption in population ecology, allowing us to aggregate data from different places to produce useful outputs, which makes it more logistically feasible to study trends at larger spatial scales.

Studies often assume ecological homogeneity within entire species, attempting to draw general conclusions without considering intraspecific variability (Moran et al., 2016; Siefert et al., 2015), despite its undoubted relevance in shaping ecology and evolution (Albert et al., 2011; Siefert et al., 2015). Moreover, even studies that do consider within‐species variation may end up assuming local homogeneity, arguing with the role of locality to explain their findings as specific responses to different environments (Kemppinen & Niittynen, 2022). Grouping together individuals sourced from close places is logically reasonable, although breaking this assumption could potentially present a major obstacle to drawing accurate conclusions.

The goal of this work is to challenge local homogeneity, to debate its reliability, and to present a case study designed to show how this assumption can be tested relatively easily.

First, let us consider the main arguments in favor of assuming local homogeneity: (I) Given the short distances, it is very likely that individuals from close sites will be connected by a conspicuous genetic flux (Petit et al., 2001). This very strong *interaction* justifies considering random drift null. (II) Given the short distances, it is very likely that the environment will be similar in each site (Diniz‐Filho et al., 2003). The overall *environmental homogeneity* justifies considering its members subjected to the same kind of natural selection, rendering adaptive shift null.

Counterarguments would logically be (I) Given fragmentation and geographical complexity, Euclidean distances alone cannot suffice to assess connectivity (Hufford & Mazer, 2003). Moreover, it is possible to argue that there is no clear relationship between genetic and quantitative trait variation based on distance since natural selection can canalize phenotypes in response to similar environmental conditions (Bonnet et al., 2021; Ortego et al., 2012; Petit et al., 2001). Moreover, barriers and obstacles are very common actors in the natural world, hindering interactions at all scales (Haila, 2002). (II) Given geographical complexity, climate is likely to be heterogeneous also at local scales (Ford et al., 2013; Garcia et al., 2022; Opedal et al., 2015). Other environmental variables could also vary locally such as soil properties, which can be very important when linking species to space at small scales (Chaney et al., 2015; Ren et al., 2022). The environmental heterogeneity introduced by this patchwork of climatic paradigms could translate into localized deviations of natural selection.

In light of these counterarguments, let us consider what would logically happen if the assumption of local homogeneity were violated between individuals we decide to group together: the consequence posing the greatest risk would be an overestimation of a population's niche, and therefore, the overestimation of their survival chances in the event of a change. This could be a logical consequence of the divergence in alleles frequency that occurs when populations are subjected to locally varying selective pressures (Balkenhol et al., 2019). Moreover, when conservation is not at stake, a violated assumption of local homogeneity could also pose the risk of ignoring the existence of populations of interest that would be culturally relevant to ecology and evolution (Balkenhol et al., 2019). Failure to acknowledge differences among groups could, therefore, strongly affect our capacity to assess and provide to the needs of a population, so we believe it is crucial to understand the limits of the local homogeneity assumption.

In recent years, multiple reports of individual niche variation documented substantial intraspecific variability between populations (Costa‐Pereira et al., 2018). Reported examples of groups that show significative differences among close provenances can be found across hundreds of taxa, from plants (Linhart & Grant, 1996) to animals (Araújo et al., 2011; Bolnick et al., 2003). However, even directly observed variations cannot suffice to justify rejecting local homogeneity for our purposes. While variations in adaptive strategies have the potential to determine a population's fate, phenotypic plasticity could also be the driver behind the observed variability (Ghalambor et al., 2007). In this scenario, any observed variation would simply be the response of a common potentiality to a variegated environment. If this was the case, then considering groups separately would lead to an underestimation of the actual population's niche, a less dire yet still incorrect conclusion for which the assumption of local homogeneity would represent an improvement.

This creates a dilemma: Should we consider the groups that show significant intraspecific variability as different populations or not?

To tackle this issue, it is imperative to consider how populations are usually defined in ecology: Odum (2000) defines populations as “a group of individuals of the same species that live together in a determined area”. The concept of a “collection of individuals of a species in a defined area” is also adopted by Mills (2012) and Smith and Smith (2017), while other authors further refine the definition with the explicit addition of common time (Cunningham et al., 2007), interactions (Cain et al., 2017) and dynamics (Berryman, 2002). Regardless of the author, two concepts seem to concern ecologists the most: (I) Members of the same population should live in the same area and (II) Members of the same population should interact with each other. These concepts are intertwined in such a way that it becomes reasonable to assign common characteristics to the group.

In this context, it is reasonable to consider different groups that show significant intraspecific variability as different populations when the driver is adaptation, and it is reasonable to consider them as parts of a single population when the driver is plasticity.

Following the observation that several Mediterranean plant species from central Italy adopt different functional trait syndromes in response to the local aridity gradient (Iozia et al., 2023), we designed a Common Garden experiment with the goal to present a possible way to deal with small scale Intraspecific Trait Variability (ITV), to accurately distinguish between adaptation and phenotypic plasticity to decide whether the local homogeneity assumption is to be considered violated between the chosen sites.

Taking our considerations to the functional‐trait approach (Violle et al., 2007) we hypothesize that samples randomly taken from neighboring provenances should reasonably share similar Plant Functional Traits (PFTs) distributions in common growing conditions if groups were part of the same population. Moreover, we hypothesize that diverging populations would show an appreciable amount of ITV when grown in a common garden, reflecting the adoption of different adaptive strategies to different environmental contexts, that is, different sensibility to specific climates.

For our case study, we measured several PFTs from groups of individuals of three Mediterranean plant species (*Quercus ilex*, *Pistacia lentiscus*, and *Cistus salviifolius*) grown in a Common Garden, sourced from three provenances that can be observed diverging in our study area (Iozia et al., 2023). The questions we specifically try to answer are “can we expect the ITV observed at a local scale to only be due to phenotypic plasticity? Should we assume local homogeneity and consider each site as parts of a single population?” The null hypothesis of our study is that, when grown in the same conditions, no ITV can be observed between the sites. For each species, this would imply ITV is just due to phenotypic plasticity and local homogeneity can be safely assumed as groups can be considered as parts of the same population.

To assess ITV, we measured several PFTs from different aerial parts of the plant that we previously measured in situ for these species. Traits vary from whole plant (plant height) to leaf morphology (leaf mass per area, leaf dry matter content, leaf tissue density) to leaf anatomy (stomatal density, stomatal area index), and were chosen for their renown responsivity to drought. Plant total height is known to decrease with drought (Nunes et al., 2017), while all the other included traits mostly respond with increases: leaf dry matter content (LDMC) reliably responds to precipitation by increasing (Anderegg et al., 2021; Wilcox et al., 2021), similarly to leaf tissue density (LTD; Gratani & Bombelli, 2001) and stomatal area index (SAI; Galmés et al., 2007). Leaf mass per area (LMA) is an important acquisitive trait which usually increases with aridity (Anderegg et al., 2021), although it has been observed to rarely adopt an inverse relationship with drought (Welles & Funk, 2021), much like stomatal density (SD; Carlson et al., 2016; Guo et al., 2017). This inconsistent behavior highlights the complexity of trait‐based ecology, given each trait responds to the environment in concert with the others to adopt an appropriate strategy that natural selection will allow to thrive.

Since some PFTs are known to be more responsive than others to environmental variations (Anderegg et al., 2021), we do not expect all traits to vary at the same magnitude. Given the different functional types of plants in this study (Harley et al., 1987; Martín‐Sánchez et al., 2022; Vignola et al., 2022), we also do not expect all species to vary the same way.

Following our expectations, our observations show a complex, species‐specific behavior that can only be observed for specific PFTs. Given the strong link between functional strategies and PFTs, we conclude that when adaptive ITV is detected, we must reject the local homogeneity assumption.

## 2 MATERIALS AND METHODS

### 2.1 Study sites

Seeds were collected in three different sites of the *Latium* region, in central Italy, during the period September–December 2021.

Each sampling site consisted of a 1 km^2^ square for each one of three sites chosen along an aridity gradient, situated around the city of Rome with a mean distance of 64.4 km from each other. For this study, we referred to this scale as *local*.

Sites were originally chosen by considering every available occurrence point for each of the considered species in the region, gathered from *GBIF* (Telenius, 2011). Then cluster analysis was set up with a 20 km radius to identify every site that shared common species composition, using the *geosphere* R package (Hijmans et al., 2017). Using a combination of aridity indexes (AI, as proposed by UNEP, 1992; Bagnouls‐Gaussen ombrothermic diagrams, Gaussen & Bagnouls, 1953) candidate sites were ranked along an aridity gradient, then the closest three sites taken from the beginning, middle, and end of the gradient were selected to maximize environmental heterogeneity while minimizing geographical distance.

**FIGURE 1:**
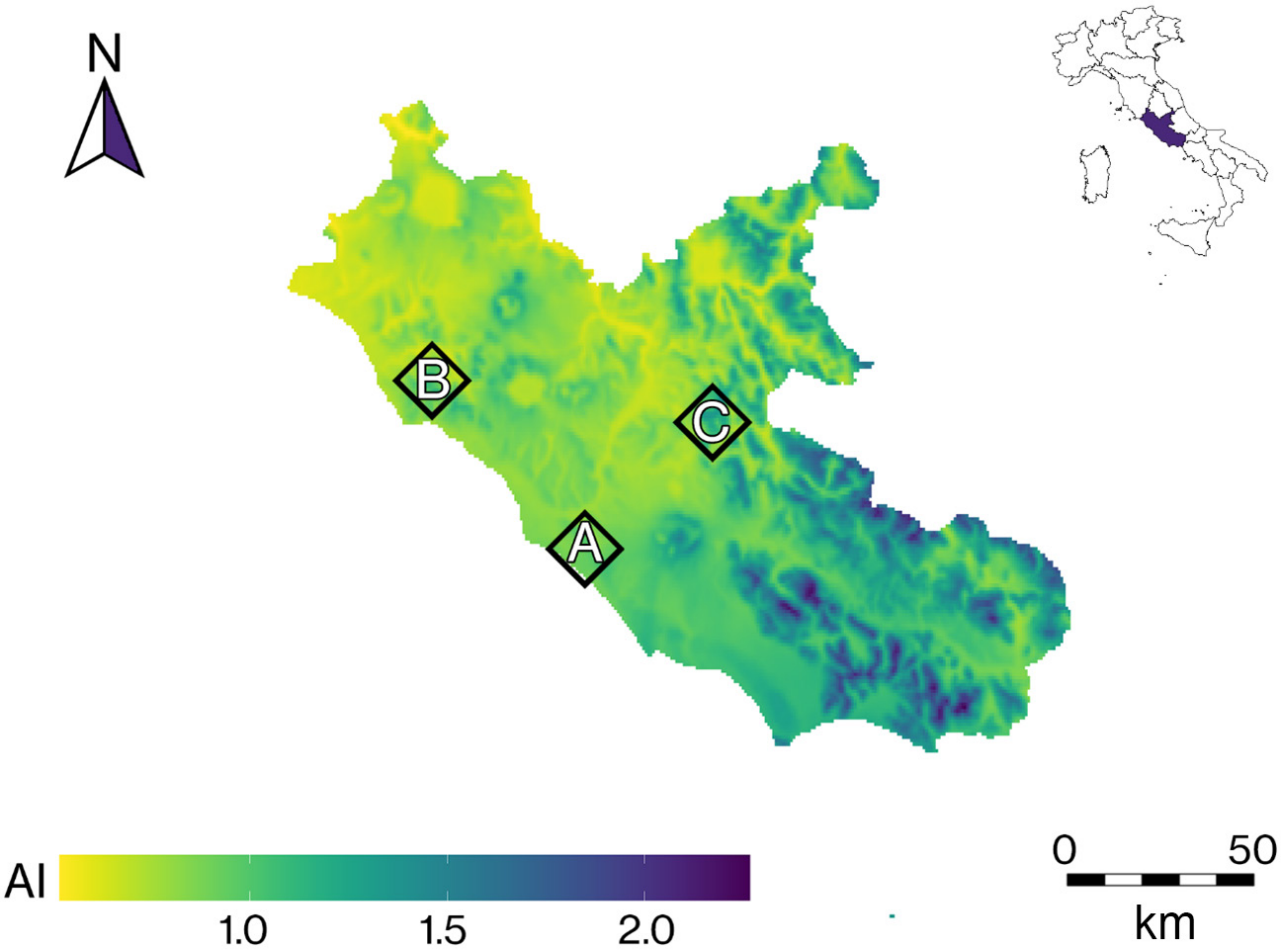
Map of the *Latium* region's Aridity Index. Site provenances are indicated as A for Castel Fusano, B for La Farnesiana and C for the Monte Catillo Natural Reserve near Tivoli. The color gradient represents the estimated Aridity Index, as presented in Iozia et al. (2023).

Climate profiles for each site were obtained with the ChelsaClimate model (Karger et al., 2017). Climate profiles were matched with data provided by the nearest meteorological stations (ARSIAL, 2022). Soils were also analyzed according to the methodologies suggested by the National Pedological Observatory of Agricultural, Food and Forest Resources Ministry (Jones Jr., 1999, data available in Iozia et al., 2023). Site A (Figure 1a) was *Castel Fusano* (3 m asl, 41°43′23.6″ N, 12°19′55.7″ E), a seaside location characterized by sandy (~90% sand) soils and a mean temperature of 16.0°C, a mean maximum temperature of 22.3°C, a mean minimum temperature of 9.9°C, and a mean total annual rainfall of 847.7 mm. This is the study's driest site. Site B (Figure 1b) was *Tenuta La Farnesiana* (150 m asl, 42°11′38.9″ N, 11°52′33.1″ E), a hilly location characterized by mostly sandy soils (~70% sand) and a mean temperature of 15.1°C, a mean maximum temperature of 21.4°C, a mean minimum temperature of 9.5°C, and a mean total annual rainfall of 938.0 mm. This is the study's second driest site. Site C (Figure 1c) was the *Monte Catillo* Natural Reserve near Tivoli (430 m asl, 41°57′51.5″ N, 12°48′54.9″ E), a mountain location characterized by sandy clay soils (~50% sand) and a mean annual temperature of 13.9°C, a mean maximum temperature of 22.1°C, a mean minimum temperature of 8.1°C, and a mean total annual rainfall of 1306.1 mm. This is the study's wettest site.

### 2.2 Study species

Three Mediterranean plant species commonly found co‐occurring in the *Latium* region were considered: *Quercus ilex* L. (holm oak), *Pistacia lentiscus* L. (lentisk), and *Cistus salviifolius* L. (sage‐leaved rock rose).

While they all share a common environment, each of these species represents a different approach to cope with arid climates.

From a functional type point of view these plants can further be divided into evergreen sclerophyllous (*Q. ilex* and *P. lentiscus*; Martín‐Sánchez et al., 2022; Vignola et al., 2022) and drought semi‐deciduous (*C. salviifolius*; Harley et al., 1987), as the first two species depend on thicker leaves and deeper roots to adopt a conservative strategy (Martín‐Sánchez et al., 2022) while *C. salviifolius* avoids drought by switching between thicker and thinner leaves every 4–8 months (Grant et al., 2014).

Evolutionarily speaking, we can appreciate yet another layer of separation: *C. salviifolius* is an indigenous taxa that evolved relatively recently under a Mediterranean climate, while the *Pistacia* genus arrived early on from the semi‐arid steppes of Central Asia (Blondel & Aronson, 1999). *Q. ilex* is usually considered a Mediterranean taxon, although recently published data seem to support a pre‐Mediterranean origin for this species (Martín‐Sánchez et al., 2022).

The last layer of separation we want to consider comes from ecology: these species differ in the successional stage at which they are dominant. *Cistus* is a pioneer species that needs direct sunlight to thrive, favors glades and is characterized by the need to be subjected to very high heat to germinate (Grant et al., 2014; Trabaud & Oustric, 1989); this adaptation allows the plant to colonize clearings opened by the intense fire regime that characterizes the Mediterranean shrubland. Lentisk becomes more competitive at later stages, usually taking over dominance at the bush phase. Although fairly resistant, this plant is also quite resilient: after settlement, lentisk can survive fires by rapidly re‐growing after an almost complete destruction of its aerial parts (Clemente et al., 2005). At climax, it is time for the extremely plastic *Quercus* trees to become dominant, trading resilience for resistance and competitiveness (Poissonet et al., 1978).

### 2.3 Seed collection and cultivation

Study sites were visited during the period June‐December 2021, to ensure complete fruit ripeness. For each site and for each species, twenty plants were visited, and similar quantities of seeds were collected from each individual.

*Cistus* seeds were extracted by sieving crushed dry fruits and stored in dry condition at 5°C. Lentisk fruits were divided into color categories, as darker fruits are considered more viable (García‐Fayos & Verdú, 1998). Seeds were extracted manually from freshly picked fruits and stored at cold, dry condition (5°C). *Quercus* acorns were floated and checked for parasites following Bonito et al. (2011). Clean acorns were stored in moist sand at 5°C. Before sowing, seed mass was measured to assess potential sources of variation between provenances, and seed mass was found to vary between sites only for Castel Fusano, where *Q.ilex* acorns were significantly bigger and heavier, *C. salviifolius* seeds were significantly lighter and *P. lentiscus* seeds were significantly smaller and lighter (data not shown). To induce germination, *C. salviifolius* seeds were submerged in boiling water for 9 minutes right before planting, following indications by Trabaud and Oustric (1989).

Seeds were planted at the experimental garden of Sapienza University of Rome (42 m asl, 41°54′8.07″ N, 12°31′2.65″ E) in 8 L plastic pots with a 4:1 ratio of soil and expanded clay (Ondoño et al., 2015). Pots were watered twice a week to maintain moisture.

The experimental site's climate was obtained with the measurements from the nearest meteorological station in the period 2006–2022 (32 m asl, 41°55′15.08″ N, 12°31′24.60″ E, ARSIAL, 2022): the garden is characterized by an annual mean temperature of 17.1°C, a mean maximum temperature of 32.8°C, a mean minimum temperature of 4.0°C, and a mean total annual rainfall of 797.5 mm. Temperatures were also recorded hourly at the growing site with a data logger (HOBO UX100‐011A Temp/RH, 2.5%; Onset). During the experiment, the garden mean temperature was 21.2°C, the garden mean maximum temperature was 32.2°C, and the garden mean minimum temperature was 12.0°C and our on‐site measurements were comparable to those obtained from the nearest meteorological station during the same period (±0.2°C).

Measurements were carried out in June 2022, following the complete germination of seedlings (i.e., the full expansion of true leaves).

### 2.4 Trait data sampling

Measurements were carried out on fully expanded leaves that developed in April 2022, after the full development of cotyledons. A total of 10 healthy individuals for each species and for each site were randomly chosen, and for each individual two leaves were sampled. For each species, this amounted to a total of 16 leaves, 20 per site, that were used to carry out morphological analyses. To carry out anatomical analyses, for each species and for each site a total of five fully expanded leaves were randomly selected from the entire pool of individuals, amounting to a total of 15 leaves per species. PFTs were measured following the same procedures as Iozia et al. (2023) to ensure comparability.

Fresh leaf area (LA, cm^2^) was measured using the image analysis system Delta‐T Devices (0–100 ± 0.1 cm^2^), and leaf thickness (LT, mm) was measured using a digital micrometer (Kennedy 331‐301, 0–25 ± 0.1 mm; Mitutoyo, JP). Leaves were hydrated until saturation for 48 h at 5°C in dark conditions, in order to measure leaf water saturated mass (SM, mg). Leaves were then dried in a thermostatic oven (M710 Thermostatic Oven; Galli, IT) at 90°C until constant weight was reached, and leaf dry mass (DM, mg) was recorded. Weights were measured with a digital balance (JK – 180, 0–180 g ± 0.1 μg; Chyo, JP).

Leaf mass per area (LMA, mg cm^−2^) was calculated as the ratio between DM and LA (Larcher, 2003), leaf dry matter content (LDMC, mg mg^−1^) as the ratio between DM and SM (Pérez‐Harguindeguy et al., 2016) and leaf tissue density (LTD, mg cm^−^3) as the ratio between LMA and LT (Pérez‐Harguindeguy et al., 2016).

Abaxial stomatal density (SD, no of stomata mm^−2^) was measured observing transparent polish impressions on the lower leaf page with an optical microscope as described by Sack et al. (2003), covering an area of 220 × 165 μm^2^. A total of five stomata for each impression were also measured in length using a digital imaging software (AxioVision AC, Release 4.5), in order to calculate the stomatal area index (SAI, no of stomata mm^−1^, following Galmés et al., 2007).

Total plant height (H, cm) was directly measured with a ruler.

### 2.5 Statistical analysis

To ensure a cautious approach, trait data were processed to remove outliers and normalize distributions. Outliers were identified through the Dixon test, which performs well with few values and breaches of normality (Dean & Dixon, 1951). When detected, observations that were over 1.5 times the interquartile range below quartile 1 or above quartile 3 were considered outliers and thus removed from the analysis.

Subsequently, trait distributions were tested for normality with the Shapiro–Wilk test. Each trait was considered separately, and contextually normalized: we used the square root for H and the inverse transformation for LDMC and LTD. Homoscedasticity was checked using Bartlett's test.

Given the goal to detect variations among sites, a proper way to answer our question is to use the One‐way ANOVA. In presence of heteroscedasticity, we adopted Welch's modification of this test. To further explore the source of variation, we followed up with a series of unpaired pairwise Student's *t* with Welch's modification.

Then we quantified the magnitude of variation among sites by measuring the Effect Size (Sullivan & Feinn, 2012). Effect Size was used to confront traits between sites in pairs, using Hedge's *G* for small sample sizes in case of equal variances and Glass's *Δ* in case of unequal variances between groups.

Statistical data processing was carried out using the R 4.0.3 statistical analysis software (R Core Team, 2022); the Dixon test was run using the R package outliers 0.15 (Komsta, 2011). Effect Size was measured using the R package effectsize 0.8.2 (Ben‐Shachar et al., 2021).

## 3 RESULTS

### 3.1 Variations among sites

**FIGURE 2:**
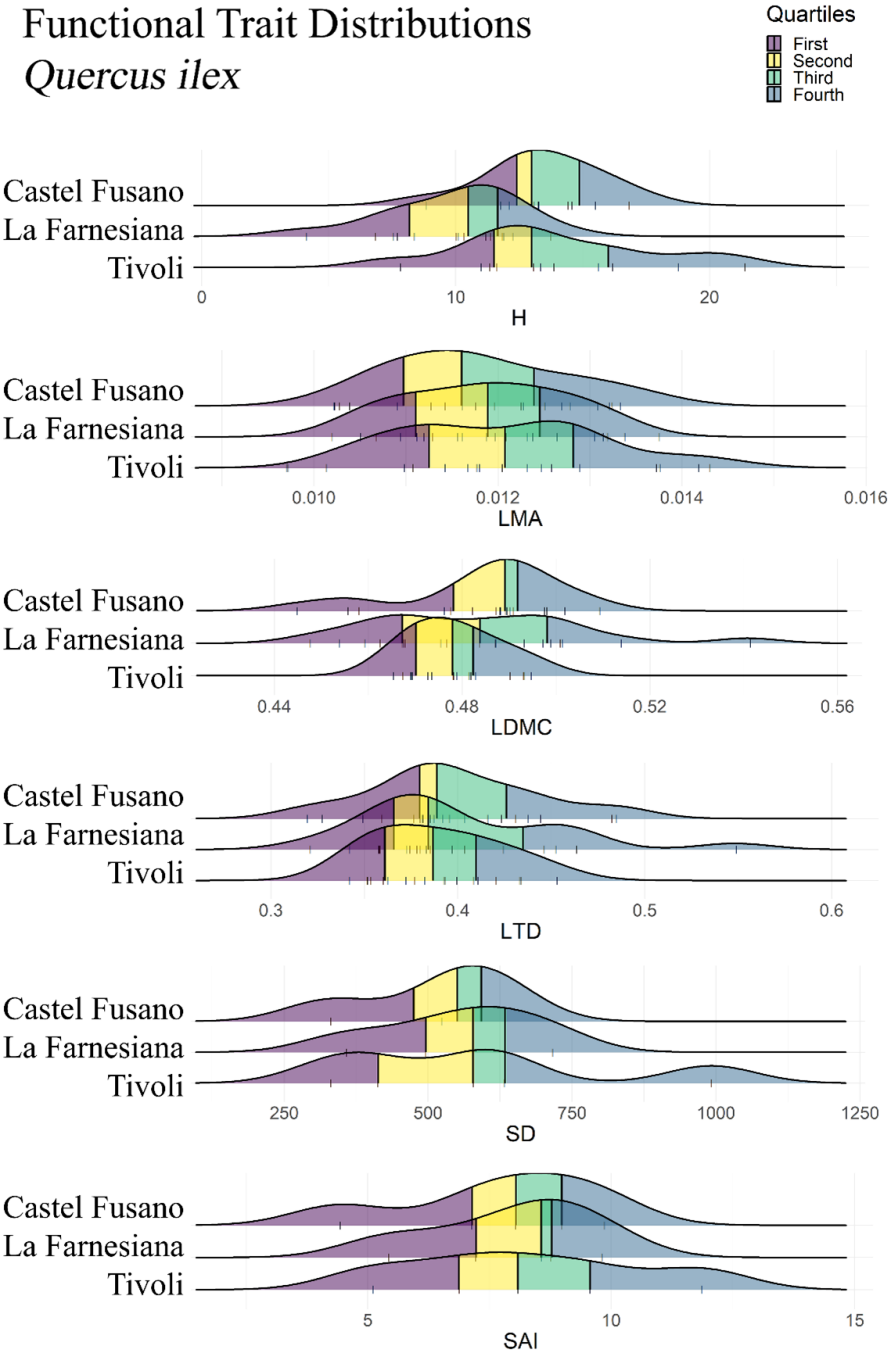
Functional Trait Distributions for *Quercus ilex*. Each ridgeline represents the density plot of the relative functional trait measured from individuals sourced from the indicated site. Original data points are visualized by vertical bars, and quartiles are represented by colors: dark blue for the first quartile, yellow for the second quartile, green for the third quartile and light blue for the fourth quartile. H, whole plant height; LDMC, leaf dry matter content; LMA, leaf mass per area; LTD, leaf tissue density; SAI, stomatal area index; SD, stomatal density.

**FIGURE 3:**
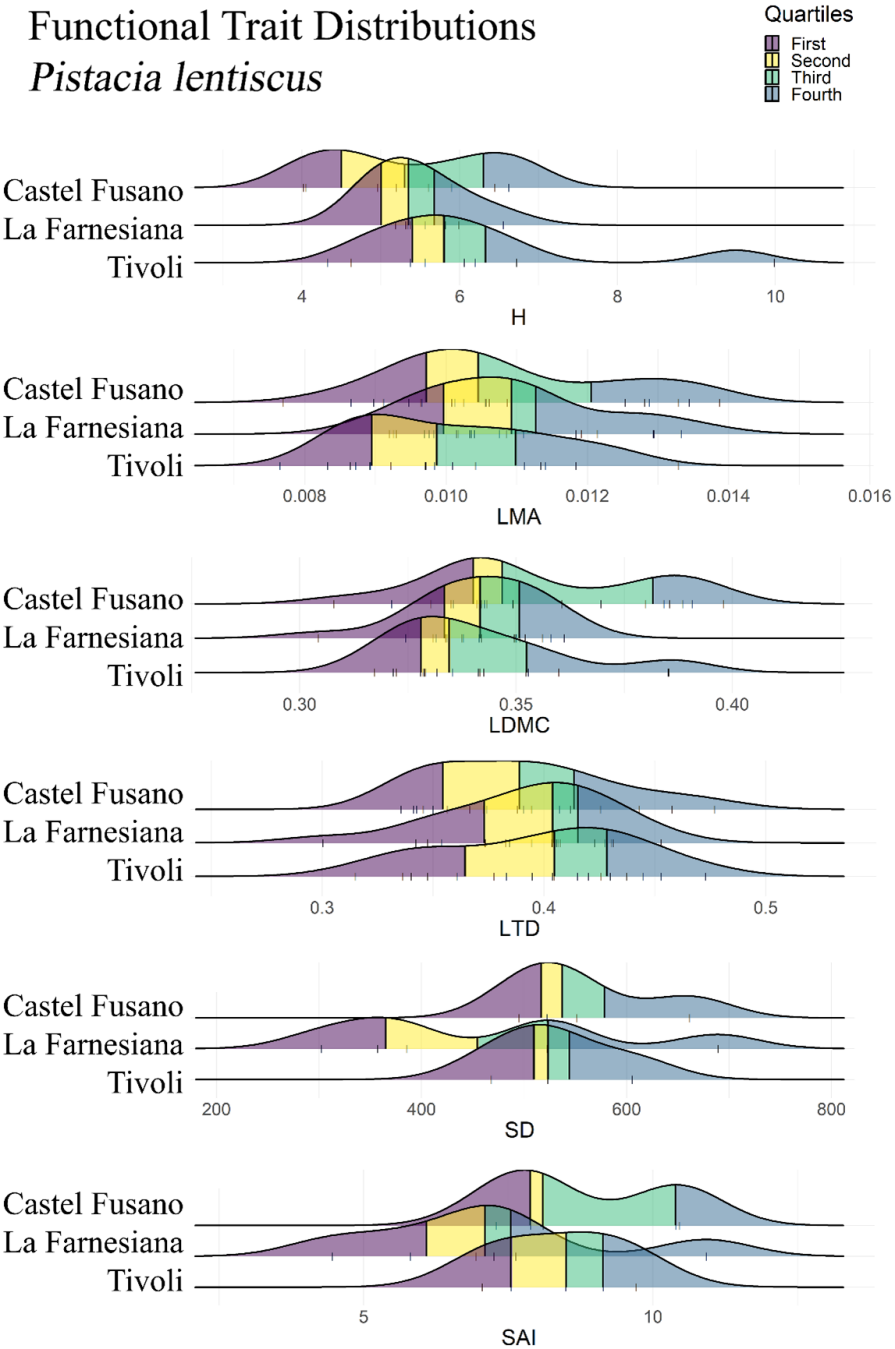
Functional Trait Distributions for *Pistacia lentiscus*. Each ridgeline represents the density plot of the relative functional trait measured from individuals sourced from the indicated site. Original data points are visualized by vertical bars, and quartiles are represented by colors: dark blue for the first quartile, yellow for the second quartile, green for the third quartile and light blue for the fourth quartile. H, whole plant height; LDMC, leaf dry matter content; LMA, leaf mass per area; LTD, leaf tissue density; SAI, stomatal area index; SD, stomatal density.

**FIGURE 4:**
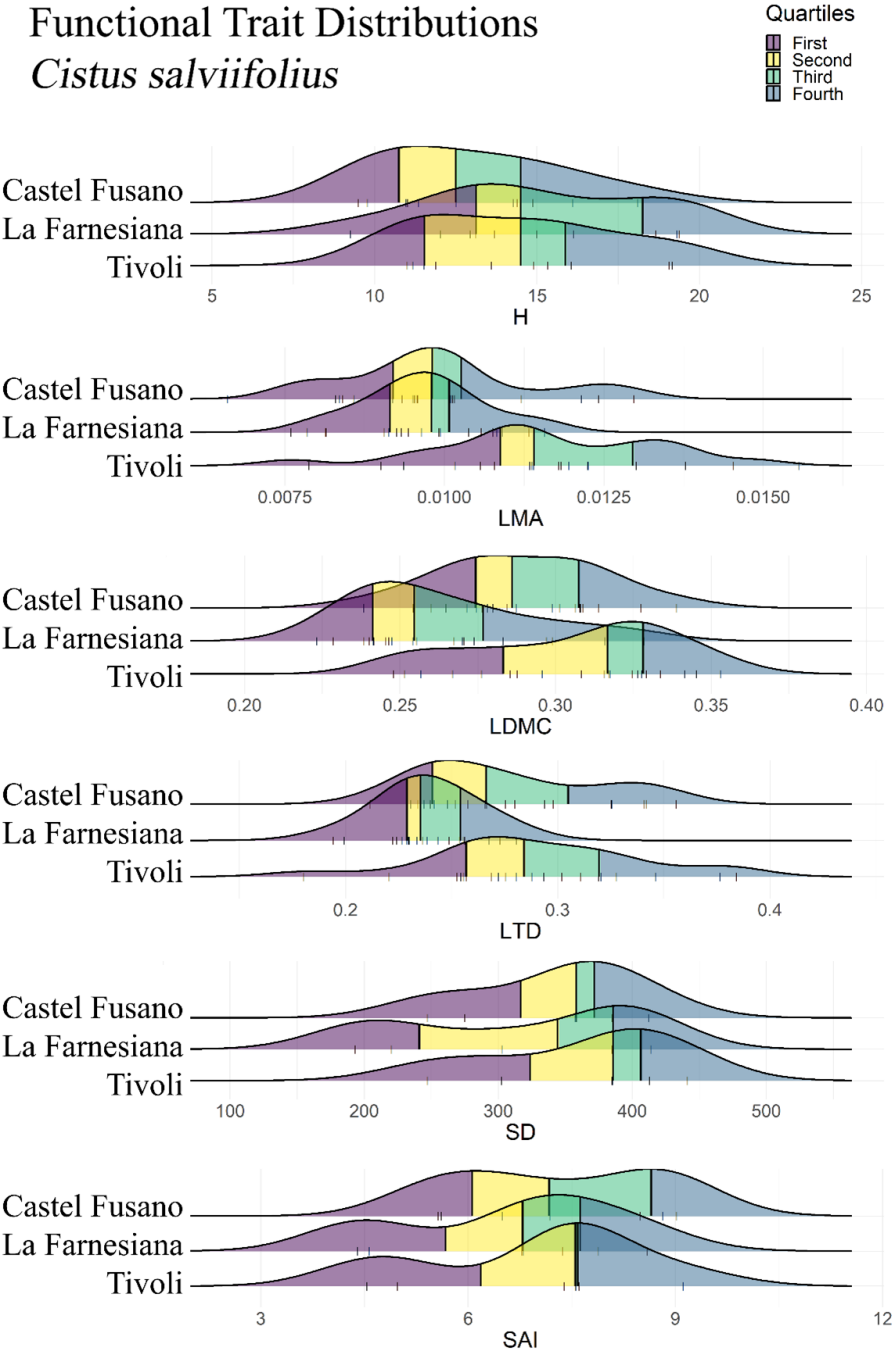
Functional Trait Distributions for *Cistus salviifolius*. Each ridgeline represents the density plot of the relative functional trait measured from individuals sourced from the indicated site. Original data points are visualized by vertical bars, and quartiles are represented by colors: dark blue for the first quartile, yellow for the second quartile, green for the third quartile, and light blue for the fourth quartile. H, whole plant height; LDMC, Leaf Dry Matter Content; LMA, Leaf Mass per Area; LTD, Leaf Tissue Density; SAI, Stomatal Area Index; SD, Stomatal Density.

Trait measurements for *Quercus ilex* obtained in the Common Garden produced similar distributions across site provenances, although individuals sourced from La Farnesiana were significantly shorter (*p*‐value = .0050; Figure 2). Similar trends were adopted by *P. lentiscus* PFTs, for which only stomatal traits seemed to differ (Figure 3), although not significantly (SD *p*‐value = .4597, SAI *p*‐value = .3900; Table 1), slightly reducing the stomatal count at La Farnesiana (Figure 3). Conversely, leaf density traits (LMA, LDMC, and LTD) of *Cistus salviifolius* markedly adopted different distributions, showing higher LMA in Tivoli (*p*‐value < .0001; Table 1, Figure 4), lower LDMC at La Farnesiana (*p*‐value = .0004; Table 1, Figure 4) as well as lower LTD from this same provenance (*p*‐value = .0003; Table 1, Figure 4).

**TABLE 1:** Results from the ANOVA performed on measurements obtained ex situ in a common garden.

Species	PFT	*F*‐value	*p*‐Value
*Quercus ilex*	**H**	**6.9470**	**.0050**
	LMA	0.3231	.7260
	LDMC	0.8037	.4573
	LTD	0.2613	.7715
	SD	0.1703	.8468
	SAI	0.0768	.9268
*Pistacia lentiscus*	H	0.8742	.4420
	LMA	2.1462	.1319
	LDMC	2.0982	.1385
	LTD	0.1414	.8686
	SD	0.8678	.4597
	SAI	1.0553	.3900
*Cistus salviifolius*	H	1.2948	.2978
	**LMA**	**9.3371**	**.0006**
	**LDMC**	**9.5412**	**.0004**
	**LTD**	**10.0360**	**.0003**
	SD	0.4267	.6642
	SAI	0.3294	.7257

*Note*: Functional traits are highlighted in bold whenever *p*‐value < .05.

Abbreviations: H, whole plant height; LDMC, leaf dry matter content; LMA, leaf mass per area; LTD, leaf tissue density; PFT, plant functional trait; SAI, stomatal area index; SD, stomatal density.

Among all species, *P. lentiscus* was the only one that failed to prove to be significantly different between sites (Table 1), while both *Q. ilex* (H; *p*‐value < .01) and *C. salviifolius* (LMA, LDMC, LTD; *p*‐value < .001) were found to adopt significantly different PFT distributions despite the common growing conditions.

A direct comparison between these data and the data published in Iozia et al. (2023) (Table 2) allowed us to further explore how these PFTs behaved between our study sites. In situ, *Q. ilex* varied significantly in leaf density traits (LMA and LDMC; *p*‐value < .02), while *P. lentiscus* showed variations at all levels, from whole plant (H, *p*‐value < .0001), to leaf density (LDMC; *p*‐value = .0096) to leaf anatomy (SD; *p*‐value = .0435) and *C. salviifolius* showed a more diversified approach to climates by significantly differing in both leaf density (LMA, LDMC; *p*‐value < .002) and plant height (H; *p*‐value = .0025). Overall, the amount of PFTs that in situ used to show significant variations among sites diminished in the generation grown under common environmental conditions, but some PFTs retained their divergences, nonetheless.

**TABLE 2:** Results from the ANOVA performed on measurements obtained in situ, as described in Iozia et al. (2023).

Species	PFT	*F*‐value	*p*‐value
*Quercus ilex*	**H**	**13.9430**	**4.71E‐05**
	**LMA**	**19.9320**	**1.24E‐06**
	**LDMC**	**4.3484**	**.0201**
	LTD	2.6064	.0870
	SD	1.8948	.1816
	SAI	1.0477	.3720
*Pistacia lentiscus*	**H**	**27.8340**	**4.85E‐08**
	LMA	0.7255	.4907
	**LDMC**	**5.3127**	**.0096**
	LTD	1.1636	.3237
	**SD**	**3.7542**	**.0435**
	SAI	1.0519	.3700
*Cistus salviifolius*	**H**	**7.0517**	**.0025**
	**LMA**	**12.6710**	**6.30E‐05**
	**LDMC**	**6.9710**	**.0029**
	LTD	2.7126	.0799
	SD	0.0369	.9639
	SAI	0.8915	.4293

*Note*: Functional traits are highlighted in bold whenever *p*‐value < .05.

Abbreviations: H, whole plant height; LDMC, leaf dry matter content; LMA, leaf mass per area; LTD, leaf tissue density; PFT, plant functional trait; SAI, stomatal area index; SD, stomatal density.

### 3.2 Size of variation

To properly understand the magnitude of the observed divergences, each pair of provenances were compared independently.

**FIGURE 5:**
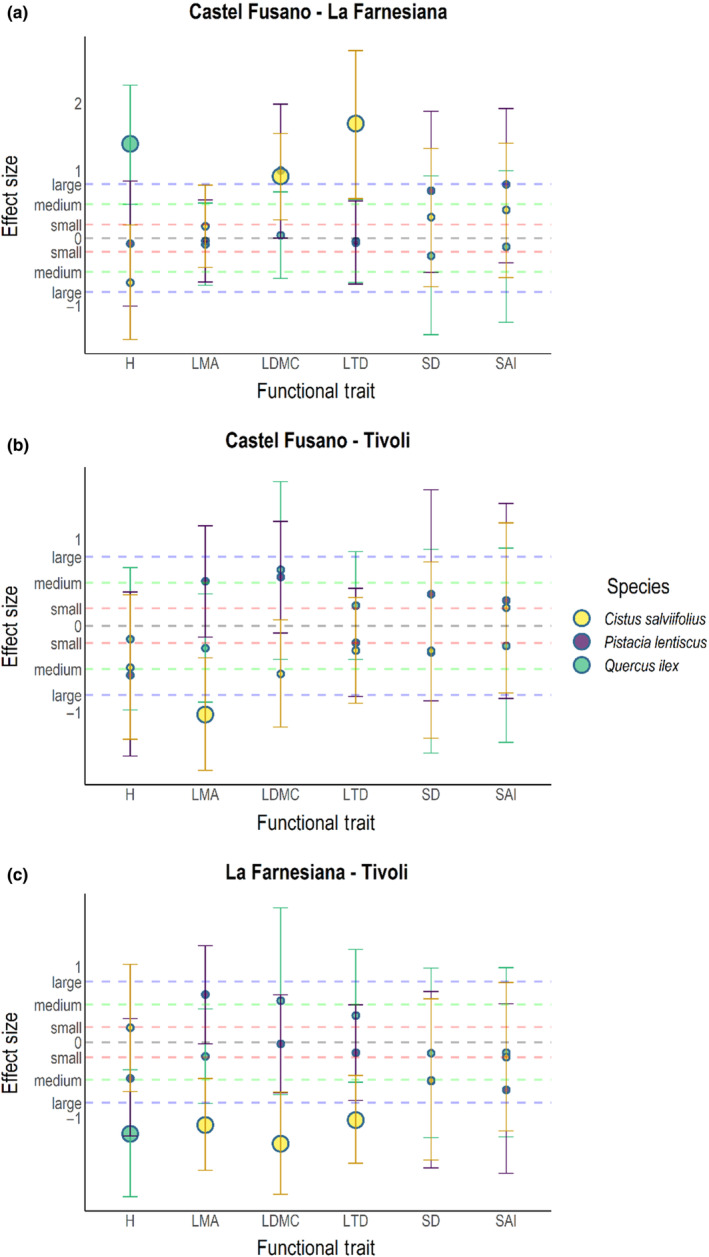
Effect Size plots for each site–site combination, obtained from measurements performed ex situ in a common garden. Points represent effect size measured with either Hedge's *G* or Glass's *Δ*, in case variances between groups are, respectively, equal or unequal. Bars represent confidence intervals. Species are represented with different colors: *Quercus ilex* is green, *Pistacia lentiscus* is blue and *Cistus salviifolius* is yellow. Dotted lines represent relative size categories as suggested by Cohen (1988). H, whole plant height; LDMC, Leaf Dry Matter Content; LMA, Leaf Mass per Area; LTD, Leaf Tissue Density; SAI, Stomatal Area Index; SD, Stomatal Density.

**FIGURE 6:**
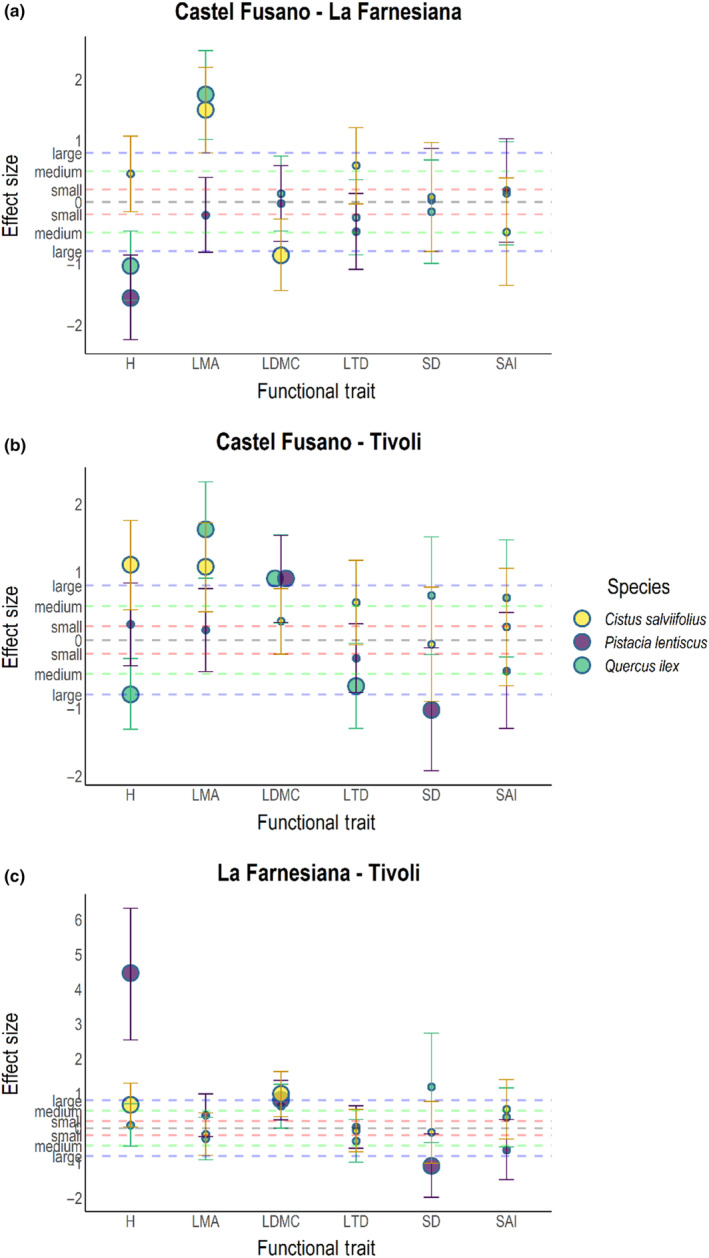
Effect Size plots for each site–site combination, obtained from measurements performed in situ, as described in Iozia et al. (2023). Points represent effect size measured with either Hedge's *G* or Glass's *Δ*, in case variances between groups are, respectively, equal or unequal. Bars represent confidence intervals. Species are represented with different colors: *Quercus ilex* is green, *Pistacia lentiscus* is blue and *Cistus salviifolius* is yellow. Dotted lines represent relative size categories as suggested by Cohen (1988). H, whole plant height; LDMC, Leaf Dry Matter Content; LMA, Leaf Mass per Area; LTD, Leaf Tissue Density; SAI, Stomatal Area Index; SD, Stomatal Density.

When we compared the driest sites (Castel Fusano and La Farnesiana, ~60 km from each other), traits that varied significantly between sites (H for *Q. ilex*; LDMC and LTD for *C. salviifolius*) always showed a *large* amount of variation (>0.8, following Cohen's relative size categorization; Cohen, 1988): Hedge's *G* for *Q. ilex*'s H was 1.394, while Hedge's *G* for *C. salviifolius*' LDMC was 0.915 and Glass's *Δ* for this species' LTD was 1.697 (Figure 5a). A similar size of variation could also be observed in situ (Figure 6a) for most of these traits, although the direction of variation was oftentimes different.

Moving on to the comparison between the driest (Castel Fusano) and the wet site (Tivoli), located at roughly 50 km from each other (Figure 5b), the main effect we could observe was in the LMA of *C. salviifolius*, where this trait showed a *large* effect size (Hedge's *G* = −1.029). This observation is substantially different from the comparison between these same sites that we could appreciate in situ (Figure 6b): all species used to show mostly large variations and *C. salviifolius*' LMA showed a different direction of variation.

The last comparison between La Farnesiana and Tivoli (Figure 5c), which are located at roughly 80 km from each other, highlighted the main differences observed in this study: *Q. ilex*'s H and *C. salviifolius*' leaf density traits (LMA, LDMC, and LTD) all shared a *large* effect size; Hedge's *G* for *Q. ilex*'s H and *C. salviifolius*' LDMC were, respectively, −1.215 and −1.345, while Glass's *Δ* for *C. salviifolius*' LMA and LTD were, respectively, −1.098 and −1.031. The direction of variation for *C. salviifolius* was again opposite to the direction shown in the comparison between the data collected in situ for these sites (Figure 6c). Interestingly, this last comparison also showed the largest effect size between all groups, for *P. lentiscus*' H observed in situ (Glass's *Δ* = 4.458).

## 4 DISCUSSION

Before moving to any action, the main concern of any study should be to demonstrate that each of its populations in examination are intrinsically homogeneous. While local homogeneity is often simply assumed, it can be addressed by demonstrating that ITV is evenly distributed within the population. In our case study, an area of the *Latium* region that includes three different provenances serves as the candidate population to test, and local homogeneity relies on the absence of any significant variation of PFTs distributions between provenances when measured in a common garden (H0).

Looking for heritable ITV is necessary since in situ traits are also subjected to phenotypic plasticity (Ghalambor et al., 2007), which could drive significant ITV without the need for adaptation. The maintenance of between‐groups ITV in a common garden represents instead a strong challenge to the assumption of local homogeneity because it demonstrates individuals from each site respond differently to the same environment.

Our case study provides evidence that there are indeed significant differences in PFTs distributions between the examined sites, rejecting the null hypothesis for at *Q. ilex* and *C. salviifolius* at this geographical scale (i.e., each site should be considered a different population), but not for *P. lentiscus* (for which is safe to consider all sites as parts of the same population).

A more precise approach is to consider which traits are essentially adapting: a direct comparison between our data and the data presented by Iozia et al. (2023) reveals that each of the considered species presents a different scenario.

In the case of *Q. ilex*, whole plant height (H) behaves as an adaptation between provenances: it shows significant differences between groups both in situ and ex situ. This is different from what happens with leaf density traits (LMA, LDMC) that showed significant variations in situ which disappeared when plants were cultivated in a common environment. This can be considered an example of phenotypic plasticity, as leaf density is driven for this species to vary in response to climate but will adopt substantially similar PFT distributions when this climatic difference disappears.

*C. salviifolius* presented us with another example of a species adapting certain PFTs while also relying on phenotypic plasticity to respond to environmental challenges with other functional traits, but here the groups of traits that were plastic or adapted were switched in comparison to *Q. ilex*. For *C. salviifolius*, plant height only showed significant ITV in situ, while leaf density traits (LMA, LDMC, and LTD) measured in the common garden were observed to adopt significantly different distributions ex situ, behaving as a local adaptation.

When present, these variations showed comparable and large effect sizes, allowing us to argue that these ITVs could potentially affect the functional strategies embraced by each population.

Interestingly, plasticity seems to be the main behavior shown by *P. lentiscus*, where none of the PFTs that showed significant ITV in situ (H, LDMC, SD) were found to vary significantly in the common garden. This is coherent with literature, as previous observations of ITV for this species at a relatively similar spatial scale in Israel and Cyprus found most of the observed trait variation to be due to phenotypic plasticity (Nahum et al., 2008).

A challenge that rises from our observations is to explain why some species adapt certain PFTs to different environments and some do not. *C. salviifolius* and *Q. ilex* have both evolved relatively recently, while *P. lentiscus* evolved in the semi‐arid steppes of central Asia (Blondel & Aronson, 1999; Martín‐Sánchez et al., 2022). Stronger plastic capabilities may have helped this species survive through the centuries and the climate change. Alternatively, plants at later seral stages might benefit from plasticity more than pioneer species, which are often found to occupy very specific niches within the community (Vandermeer, 1996). These are all possibilities that could be explored by future studies.

It is also interesting to consider the direction of variation within sites, both in situ and ex situ. Counterintuitively, while in situ each species behaved as expected from literature (Anderegg et al., 2021; Carlson et al., 2016; Nunes et al., 2017), responding with conservative traits (i.e., lower height, thicker leaves) to drier environments, ex situ the tables turned toward Tivoli, the “wet” provenance, driving plants to adopt more conservative strategies than the other groups. A possible explanation would be that plants adapted to the rainy conditions of Tivoli might perceive the environment of the common garden as a source of stress more than the others, so it would respond with the adoption of a stress response. It is worth noting in this case that each PFT from the dry sites showed a less conservative mode in the common garden, reflecting the perception of a less stressful environment than nature for these plants.

Explaining these responses becomes a complex task if we also consider more factors that may be driving these changes between sites. While aridity is a major factor shaping these sites, we must also note that soils, height, distance from the sea and biotic communities are different between sites. This constitutes a limitation if our intent is to assess which population might be more affected by climate change, but the common garden setup might also solve this problem by performing a water stress experiment in which we can also simulate drought and assess its physiologic effect on plants. A substantial divergence in the effectiveness of adaptive strategies could constitute an even stronger argument against the assumption of local homogeneity. Evidence supporting the conclusion that plants from neighboring provenances grown in a common garden show a different physiological response to stressful climates would add another layer of nuance and importance to the choice of rejecting local homogeneity, as we would be implying these populations should also be considered as ecotypes (Lowry, 2012). We reckon obtaining this kind of evidence is challenging, and while desirable we do not deem it necessary for the purpose of choosing whether to assume local homogeneity or not.

Overall, our findings provided us with the opportunity to show how this conceptual framework can drive decisions toward both the adoption and the rejection of the local homogeneity assumption, depending on which species and traits we wish to consider.

Our observation that H and leaf density traits (LMA, LDMC, and LTD) are often involved in either adaptive or plastic responses while stomatal traits tend instead to be more conserved within our region is coherent with the common knowledge that PFTs show diverse responses to environmental variations (Anderegg et al., 2021; Carlson et al., 2016; Nunes et al., 2017), and allows us to appreciate that our conclusions may diverge basing on which traits we are considering to choose whether to assume local homogeneity.

If any of these PFTs could potentially affect survival strategies (Violle et al., 2007), we argue that it may be reasonable to consider a wide set of traits and to reject the local homogeneity assumption at the emergence of any significant adaptive divergence between the groups. Potential gains from this approach can be already found in literature, as there are several examples of research that can show how considering local heterogeneity could lead to useful outcomes, from ecological restoration (Bischoff et al., 2006) to population ecology (Myers et al., 2019; Penaluna et al., 2015), and even behavioral ecology (Dubuc‐Messier et al., 2017).

In summary, our results highlight how easily it is possible to challenge and break the assumption of local homogeneity for most of the considered species, providing us with new directions in which to focus our research. This shows the importance of testing for local variability, with the demonstration that even a small spatial scale (in the range of tens of Kilometers) might be appropriate to define populations of *P. lentiscus* but inappropriate to ensure local homogeneity for *Q. ilex* and *C. salviifolius*, adding yet more urgency to take into consideration ITV within our studies. This is made more difficult by the relative paucity of literature assessing ITV at local scales (Albert et al., 2011; Moran et al., 2016; Siefert et al., 2015), yet looking for evidence of local variation and focusing on the difference between plasticity and adaptation could be the appropriate tool to exploit when defining our populations.

## AUTHOR CONTRIBUTIONS

**Lorenzo Maria Iozia:** Conceptualization (equal); data curation (lead); formal analysis (lead); funding acquisition (equal); investigation (equal); methodology (supporting); visualization (lead); writing – original draft (lead); writing – review and editing (equal). **Laura Varone:** Conceptualization (equal); investigation (equal); methodology (lead); project administration (lead); supervision (lead); writing – review and editing (equal).

## FUNDING INFORMATION

This research was funded by PhD School XXXVI Cycle, Sapienza University of Rome.

## CONFLICT OF INTEREST STATEMENT

No conflict of interest declared.

### OPEN RESEARCH BADGES

This article has earned an Open Data badge for making publicly available the digitally‐shareable data necessary to reproduce the reported results. The data is available at https://doi.org/10.5061/dryad.hqbzkh1nv.

## Data Availability

The data supporting the findings of this study are available at the Dryad public repository, with the https://doi.org/10.5061/dryad.hqbzkh1nv.
